# A cross-sectional study of blood pressure control in hypertensive patients in general practice (the I-TARGET study)

**Published:** 2009-08

**Authors:** Brian Rayner, Hermanus S Schoeman

**Affiliations:** Division of Nephrology and Hypertension, University of Cape Town, South Africa; Department of Clinical Biostatistics, Medical University of Southern Africa, Garankuwa, South Africa

## Abstract

**Introduction:**

Despite the availability of multiple effective antihypertensive drugs, hypertension control rates remain poor. The reasons for this are complex, but increasingly, physician inertia has been identified as a crucial factor. In this study we attempted to define the level of blood pressure (BP) control and reasons for not achieving control in a survey of selected general practices within South Africa.

**Methods:**

This was a multi-centre, cross-sectional disease study involving 15 selected general practices throughout South Africa. Treated hypertensive patients over 18 years old were eligible for inclusion. The study was approved by Pharma Ethics, and after informed consent, consecutive hypertensive patients at the participating general practice centres were included, with each centre enrolling 30 patients.

**Results:**

A total of 451 patients, from 15 sites in South Africa, were entered in the study. The mean age of the patients was 60.7 years, 56.3% were female and 15.7% were current smokers. The BP was reduced by 26.4/17.6 mmHg (*p* < 0.001) in 220 patients with a documented initial BP. Co-morbidities were present in 322 (71.4%) patients and overall, 37.9% had more than one co-morbidity. Lifestyle modification was not uniformly applied, with only 46.1, 59.6 and 56.8% receiving advice about weight loss, exercise and diet, respectively. Less than a third (30.7%) of patients were on monotherapy, 42.8% were on two drugs (25.9% on fixed-drug combination and 16.9% on free combination) and 26.5% were on more than two agents. Most (86.9%) practitioners used either international or local guidelines to determine target BP. Overall, 61.2% of patients were at goal (BP < 140/90 mmHg). If a stricter target BP (BP ≤ 130/80 mmHg) is applied to patients with co-morbidities, as recommended by the guidelines, 60.6% of patients did not reach goal. Of the 175 patients not at target BP, there was no action plan in 22.9%, while 39.4% were advised to undertake lifestyle changes only.

**Conclusions:**

Control rates were quite good in comparison with other surveys within and outside South Africa. However we were able to define several important deficiencies: there was evidence of physician inertia and also practitioners need to be more cognisant of local and international guidelines to optimise treatment.

## Summary

Hypertension affects more than 20% of the adult population and is the most important risk factor for heart and kidney disease, and stroke in South Africa. The South African Hypertension Guidelines recommends that the target blood pressure (BP) for hypertensive patients should be below 140/90 mmHg.^1^ However in patients at high cardiovascular risk, such as those with several risk factors, target-organ damage or complications, the target should be lowered to below 130/80 mmHg. Similar recommendations for BP control are made by the British Hypertension Society and the Seventh Report of the Joint National Committee on Prevention, Detection, Evaluation and Treatment of High Blood Pressure (JNC-7).^2^,^3^

Despite the availability of multiple effective antihypertensive drugs, control rates remain poor. In developed and developing countries, less than 27 and 10%, respectively, of hypertensive patients have achieved target BP.^3^,^4^ The reasons for the poor control rates are complex, but increasingly, physician inertia has been identified as a crucial factor.^4^ Physician inertia is the failure to implement appropriate guidelines for hypertension management and to modify antihypertensive treatment when the patient’s BP is known to be persistently above target.

## Methods

This was a multi-centre, cross-sectional disease study involving 15 selected general practices throughout South Africa. Treated hypertensive patients over 18 years old were eligible for inclusion. The study was approved by Pharma Ethics, and after informed consent, consecutive hypertensive patients at the participating general practice sites were included. Each site was expected to enrol 30 patients over a three- to six-month period, giving a total of 450 patients, and all BPs were taken by the participating practitioner. The following information was recorded in the study: patient demographics, date of first diagnosis and duration of disease, current antihypertensive treatment, baseline, initial and current BP, co-morbidities and other risk factors, medical and surgical history, current concomitant medication, target BP goal and treatment modification.

The overall objective of the study was to improve general practitioners’ management of hypertension by increasing awareness of the importance of blood pressure goal attainment in hypertensive patients, and to assess the number of patients on antihypertensive therapy that were not at target blood pressure according to the current hypertension treatment guidelines. General practitioners were not blinded to the objectives of the study.

It was estimated that between 10 and 30% of hypertensives in the study would be uncontrolled. Therefore it was estimated that 450 evaluable patients were needed to obtain a precision (α = 5%) within the proportions ranging between ± 2.5 and ± 4%. Descriptive statistics were used to generate an individual practice profile for each participating general practitioner. Arithmetic means, medians and standard deviations were calculated for numerical data as appropriate. Categorical data were summarised in frequency tables and bar charts.

## Results

A total of 451 patients from 15 sites in South Africa were entered in the study. Eight of the sites were in Gauteng, three in Kwazulu-Natal, three in the Western Cape and one in the Northwest, and all were in urban areas. Fourteen sites each recruited 30 patients and one site recruited 31 patients. All the patients entered in the study complied with the inclusion criteria. The mean age of the patients was 60.7 years, 56.3% were female and 15.7% were current smokers. The initial BP was known in 220 patients and the mean BP was 160.6/100 mmHg, and was reduced by 26.4/17.6 mmHg (*p* < 0.001 for both systolic and diastolic BP). Co-morbidities were present in 322 (71.4%) patients and overall, 37.9% had more than one co-morbidity. The distribution of these is shown in Table 1.

**Table 1:** Distribution Of Patient Co-Morbidities

*Co-morbidity*	*n (%)*
Diabetes mellitus	84 (18.6)
Metabolic syndrome	77 (17.1)
Peripheral arterial disease	5 (1.1)
Diabetic nephropathy	5 (1.1)
History of atrial fibrillation	12 (2.7)
History of myocardial infarction	12 (2.7)
Obesity	164 (36.4)
Coronary artery disease	31 (6.9)
Elevated cholesterol	200 (44.3)
Congestive heart failure	7 (1.6)
History of stroke	11 (2.4)
Microalbuminuria	11 (2.4)

Lifestyle modification was not uniformly applied, with only 46.1, 59.6 and 56.8% receiving advice about weight loss, exercise and diet, respectively.

**Fig. 1.**
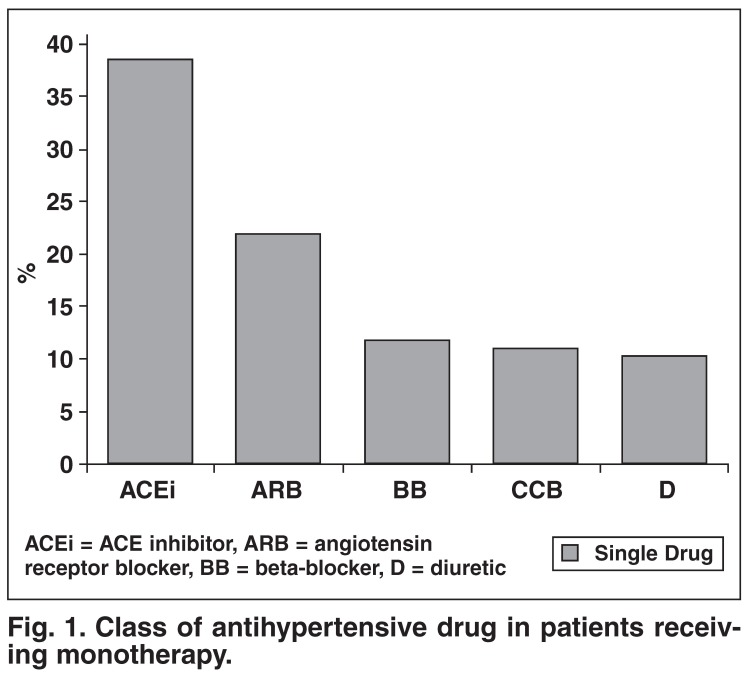
Class of antihypertensive drug in patients receiving monotherapy.

**Fig. 2.**
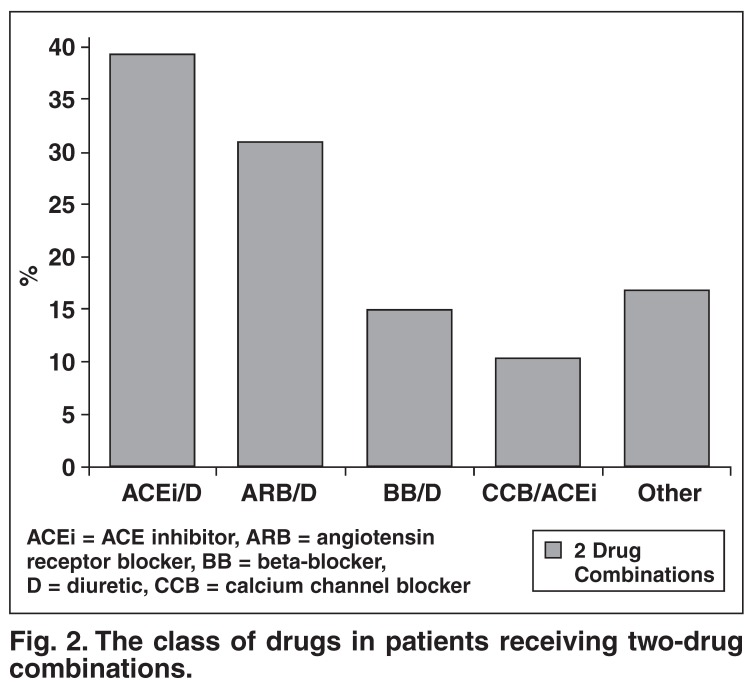
The class of drugs in patients receiving two-drug combinations.

With regard to antihypertensive therapy, 30.7% of patients were on monotherapy, 42.8% were on two drugs (25.9% on a fixed-drug combination and 16.9% on a free combination) and 26.5% were on more than two agents. The class of drug used as monotherapy and as free or fixed combinations is shown in Figs 1 and 2.

Most practitioners (86.9%) used either international or local guidelines to determine target BP. Overall, 61.2% (95% CI: 56.7–65.7%) of patients were at goal, i.e. BP < 140/90 mmHg, with a range of 30–93.3% between sites. Over half of the patients (62.3%) on monotherapy had controlled BP. Co-morbidities were present in 71.4% of the patients and the mean BP of patients with or without co-morbidities is shown in Table 2. Diastolic BP was slightly higher in patients with co-morbidities (*p* = 0.025), but there was no difference in systolic BP. However, if a stricter target BP (BP ≤ 130/80 mmHg) was applied to patients with co-morbidities, as recommended by the guidelines, 60.2% (95% CI: 55.3-65.9%) of patients did not reach target.

**Table 2:** Mean BP In Patients With Or Without Co-Morbidities

*Patient status*	*n*	*Mean*	*SD*
With co-morbidities			
Systolic BP (mmHg)	322	131.4	7.57
Diastolic BP (mmHg)*	322	83.5	5.21
Without co-morbidities			
Systolic BP (mmHg)	129	131.3	8.60
Diastolic BP (mmHg)*	129	84.7	4.68

*p = 0.025

Of the 175 patients not at target BP, there was no action plan in 22.9%, while 39.4% were advised to undertake lifestyle changes only (Table 3). In the remainder, the practitioner increased the dose, added another drug, switched medication to a fixed combination, or changed to another monotherapy.

**Table 3:** Actions Planned By Practitioners To Achieve Target BP

*Action*	n (%)
None	40 (22.9)
Increase dosage	24 (13.7)
Lifestyle modification	69 (39.4)
Add another drug	33 (18.9)
Switch medication to combination	18 (10.3)
Switch medication to another monotherapy	4 (2.3)

## Discussion

The I-TARGET study provides important information regarding the status of hypertensive care in primary private practice in South Africa. On first analysis, the relatively good news is that control rates are comparably good compared to surveys conducted in other countries and in South Africa. Less than a third (61.2%) of patients had their BP controlled to levels below 140/90 mmHg. This compared favourably to data reported by Rayner and Becker, which showed that only 47.9 and 65.5% of patients had systolic BP < 140 and diastolic BP < 90 mmHg, respectively.^5^

However, more detailed analysis of the data revealed important physician-related deficiencies in hypertensive care, even though 86.9% of practitioners used either international or local guidelines to determine overall hypertensive management and target BP. For example, if we consider more appropriate targets for high-risk patients (BP ≤ 130/80 mmHg), the picture is less promising, with 60.6% of patients not at target. This suggests that practitioners are applying a uniform target BP of 140/90 mmHg and not stratifying patients according to cardiovascular risk, and applying a lower target of ≤ 130/80 mmHg for those at high risk, according to the guidelines.

This dissociation of clinical practice and implementation of guidelines is a worldwide phenomenon, which has led to the concept of physician inertia as an important contributing factor to suboptimal care of hypertensive patients. Several other examples are identified in this survey. Lifestyle modification was not uniformly applied, with only 46.1, 59.6 and 56.8% receiving advice about weight loss, exercise and diet, respectively. This suggests a focus on drug therapy and less emphasis on lifestyle. It goes without saying, that lifestyle changes are not only important for assisting with BP control but also for lowering the chance of cardiovascular morbidity and mortality, which is the ultimate aim of a comprehensive treatment strategy.

The response of the practitioner to a BP not at target further illustrates this problem. In the 175 patients not at target BP, there was no action plan in 22.9%, despite the fact that the practitioner had self identified these patients not to be at target and the majority of patients were either on one- or two-drug treatment.

Choice of antihypertensive therapy in this survey showed interesting trends. Currently, the South African Hypertension Guideline recommends that diuretics are first-line therapy for hypertension.^1^ However, in this survey, only 11.6% of patients on monotherapy were on a diuretic. The most commonly prescribed monotherapy was either an ACE inhibitor or angiotensin receptor blocker (ARB), which probably reflects more contemporary trends in hypertensive management.^6^ Although diuretics are effective, they are associated with long-term metabolic effects and poorer tolerability, whereas ACE inhibitors or ARBs are better tolerated and potentially offer better long-term protection against target-organ damage. The low level of use of β-blockers probably reflects the acceptance by practitioners that these agents are now considered fourth-line therapy in the absence of compelling indications for their use.

The use of two-drug combination therapy (either fixed or free) also showed interesting trends. The South African Hypertension Guideline recommends the following combination treatments for essential hypertension after failure of first-line therapy – diuretic plus an ACE inhibitor, ARB or calcium channel blocker (CCB), and CCB plus ACE inhibitor or ARB. Beta-blockers plus diuretic are not generally recommended, especially in patients with the metabolic syndrome, due to the long-term risk of type 2 diabetes.

Not unexpectedly, diuretics plus ACE inhibitor or ARB (57.8%) were the commonest choice most often in fixed-drug combination. Of concern, 29% of patients were receiving two-drug combinations not generally recommended, of which 13.7% were for a diuretic and β-blocker. Only 9.9% of patients received a CCB plus ACE inhibitor or ARB. In the future, this combination may be increasingly utilised because of the results of the ACCOMPLISH study, which showed a 20% reduction in cardiovascular endpoints in patients receiving a fixed CCB/ACE inhibitor combination versus a fixed diuretic/ACE inhibitor combination.^7^

There are important weaknesses to the study. The sites selected were based on convenience and all were situated in urban areas. Therefore the results cannot necessarily be generalised to the whole of South Africa. The results were also cross-sectional and may not have reflected the overall BP control of an individual patient. For instance, a practitioner may not have changed treatment in a patient with an isolated office reading slightly above normal where all previous readings had been in the target range. In addition, 24-hour ambulatory BP monitoring was not done to assess the effects of white-coat hypertension. Therefore the level of physician inertia may have been overstated in the study. Furthermore, within the limits of the study, it was not always possible to have insight into the choice of treatment in an individual patient. There may also have been a tendency to bias, as practitioners may have only entered patients with well-controlled BP. For instance in some practices, over 90% of patients were controlled, but of course this may also represent clinical excellence.

## Conclusion

The I-TARGET survey had important insights into hypertensive care in general practice. Control rates were quite good in comparison with other surveys within and outside South Africa.^4^,^5^ However, we were able to define several important deficiencies: there was evidence of physician inertia and also practitioners need to be more cognisant of local and international guidelines to optimise treatment.
